# Persistent Postural-Perceptual Dizziness (PPPD) and quality of life: a cross-sectional study

**DOI:** 10.1007/s00405-023-08040-7

**Published:** 2023-05-31

**Authors:** Malin Herwander Steensnaes, Mari Kalland Knapstad, Frederik Kragerud Goplen, Jan Erik Berge

**Affiliations:** 1 Department of Child and Adolescent Psychiatry, Helse Fonna, Haugesund, Norway; 2 Department of Otorhinolaryngology, Head and Neck Surgery, Haukeland University Hospital, Bergen, Norway; 3 Department of Clinical Medicine, University of Bergen, Bergen, Norway; 4 Department of Health and Functioning, Western Norway University of Applied Sciences, Bergen, Norway

**Keywords:** Dizziness, Quality of life, Anxiety, Depression, Questionnaire, Diagnosis, Persistent postural-perceptual dizziness, PPPD, Dizziness handicap inventory, DHI, RAND-12, Hospital anxiety and depression scale

## Abstract

**Purpose:**

To determine if Persistent Postural-Perceptual Dizziness (PPPD) is associated with increased burden of dizziness and quality of life. Secondly, if this association is present, to determine if it can be explained by differences in anxiety and/or depression between patients with PPPD and dizzy patients without PPPD.

**Methods:**

Cross-sectional study performed in an outpatient otolaryngology clinic, including patients 18–67 years referred from primary care for suspected vestibular disease with chronic dizziness. Patients underwent clinical examination and completed the following questionnaires: Dizziness Handicap Inventory (DHI), RAND-12 Health Status Inventory and Hospital Anxiety and Depression Scale (HADS). Scores in DHI and RAND-12 were compared between patients diagnosed with PPPD and patients without PPPD.

**Results:**

202 patients were included. 150 (74%) were women and 37 (18%) were diagnosed with PPPD. Patients in the PPPD group had increased burden of dizziness and reduced quality of life (QoL) as shown by a higher mean DHI score (49.2 vs. 30.8; *p* < 0.001) and reduced mean RAND-12 physical score (39.0 vs. 44.6; *p* = 0.004). After adjusting for age, gender and HADS, PPPD was associated with a 15.3 (*p* < 0.001) points increase in DHI score, and a 4.0 (*p* = 0.020) points decrease in RAND-12 physical score.

**Conclusion:**

Patients with PPPD have a higher burden of dizziness and a lower physical health-related quality of life (HRQoL) compared to other dizzy patients. The difference was evident also after adjusting for anxiety and depression, illustrating how PPPD is a different entity than these common psychiatric conditions.

## Introduction

Large population-based studies show that dizziness and vertigo are associated with reduced quality of life (QoL) and affects as much as 15–20% of adults per year, showing a particularly high prevalence and incidence among women and in the elderly population [1].

Persistent Postural-Perceptual Dizziness (PPPD) was defined by the Bárány Society in 2017 and included in the 11th edition of the International Classification of Diseases (ICD-11) [2]. The diagnosis is derived from common traits of phobic postural vertigo (PPV), space-motion discomfort (SMD), visual vertigo (VV) and chronic subjective dizziness (CSD) [3]. PPPD is a chronic and functional vestibular disorder, and patients experience symptoms of dizziness, unsteadiness or non-spinning vertigo, which is exacerbated by upright posture, active or passive movement and exposure to moving- or complex visual stimuli [4].

The association between dizziness and psychiatric comorbidity, especially anxiety and depression, is well known [1, 5–9]. PPPD may co-exist with structural and/or psychological disorders, but is defined as a different entity [10, 11]. It is not uncommon for patients to develop secondary anxiety, depression, avoidance behavior or social phobia [1, 3, 12].

Even though several studies have used the HRQoL-measures in evaluating treatment effect in patients with PPPD, there are few studies evaluating the symptom burden in patients with PPPD compared to other diagnoses. Our study aims to investigate whether PPPD is associated with an increased burden of dizziness and reduced quality of life, and if so, to determine if this association could be explained by differences in anxiety and/or depression (i.e., emotional distress) between patients with PPPD and dizzy patients without PPPD.

## Material and methods

### Study population and design

We conducted a cross-sectional study consisting of consecutive patients referred for suspected vestibular disease to an otolaryngology-clinic and symptom duration more than 3 months. Patients between 18 and 67 years referred from a primary physician to the ear, nose and throat (ENT)-clinic at Haukeland University Hospital in Bergen, Norway, were invited to participate in the study. The participants completed questionnaires, underwent vestibular testing, were examined by an otolaryngologist and a physiotherapist. The examinations were performed in the outpatient clinic. The study was approved by the Regional Committee for Medical and Health Research Ethics (REK) in Western Norway (REK number 2017/783). Written informed consent was obtained from all participants.

The questionnaires were completed prior to examination in the clinic and included the Dizziness Handicap Inventory (DHI), RAND-12 Health Status Inventory, Hospital Anxiety and Depression Scale (HADS) and general information. Patients that did not complete the questionnaires or had missing data were excluded from the analyses. Vestibular testing included posturography, videonystagmography including positional testing, bi-thermal caloric testing, in addition to audiometry. Patients were diagnosed according to ICD-10 by a treating otolaryngologist. For the purpose of this study, the diagnoses were retrospectively revised by two of the co-authors to correspond with the ICD-11. Patients who fulfilled diagnostic criteria of specific diagnoses were diagnosed with these. Patients that did not fulfill diagnostic criteria were categorized according to the time-aspect of symptoms according to the ICD-11 with either acute, episodic or chronic vestibular syndrome not further specified.

### Statistics

For statistical analyses patients diagnosed with PPPD were compared to patients with other dizziness diagnoses. Continuous data are presented as means, ranges and standard deviation. Categorical variables are reported with absolute and relative frequencies. Continuous and categorical variables were compared with student’s t-test for variables that were normally distributed and with Mann–Whitney U-test for non-normal variables that could not be transformed. Duration of dizziness was highly skewed and was therefore log-transformed prior to t-testing. Two-sided *p* values < 0.05 were considered statistically significant. Associations between DHI, RAND-12 and PPPD were examined by linear regression analyses, with DHI and RAND-12 (physical and mental domain separate) as dependent variables and PPPD as an independent variable. Age, gender and HADS score were used as adjustment variables. Logistic backwards regression analysis with PPPD as dependent variable were performed by first including all questions from the DHI and then stepwise removal of questions with *p* > 0.05 for being diagnosed with PPPD. Data were analyzed using Stata 15, StataCorp LLC 2017 (*Stata Statistical Software Release 15.* College Station, TX: StataCorp LP).

### Questionnaires

The Dizziness Handicap Inventory (DHI) [13] is a self-assessment questionnaire, consisting of 25 items that aims to evaluate the self-perceived handicap due to vertigo and dizziness. Items are divided into functional, emotional and physical subscales, with a total score range from 0 to 100. The total score is recommended when comparing scores on an international level [14]. The higher the score, the greater the challenges; a score > 29 indicates disability [13]. The score correlates with the patients’ perceived handicap due to dizziness and can be used to assess the burden of dizziness and degree of ailments in dizzy patients [15].

The RAND-12 Health Status Inventory is a short version of the RAND-36. The surveys are identical to the 36- and 12-Item Short Form Survey (SF-36 and SF-12). RAND-12 measures health-related quality of life (HRQoL) within eight concepts (physical functioning, role limitation due to both physical and emotional health problems, bodily pain, general health, vitality, social functioning and mental health) and generates the physical component summary and mental component summary scores [16]. A high score indicates a better health status and thus a higher HRQoL [17].

The Hospital Anxiety and Depression Scale (HADS) [18] is a self-assessment scale used to identify symptoms of anxiety and depression in the last four weeks, and is a measurement of emotional distress. It consists of 14 questions, with each question scaled from 0 to 3. The form is divided into two domains, anxiety and depression, with a maximum score of 21 on anxiety and depression, respectively. A total score of 11 or higher on either of the domains indicates the presence of the disorder(s), while a score of 8–10 indicates the suggestive presence of the disorder(s) [19]. We have used HADS as a 1-dimensional tool and did not use the anxiety and depression subscales; our goal was to screen for emotional distress and not to diagnose psychiatric comorbidity. This is also as recommended in a previous investigation of factor structure, internal reliability and convergent validity using HADS on dizzy patients [20]. In addition, participants filled out questionnaires regarding general health status and further details of their dizziness symptoms and duration.

## Results

A total of 227 patients met the inclusion criteria. 16 patients did not fulfill the questionnaires and 9 patients were excluded due to technical issues. Total participants included in the study were 202. Age ranged from 18 to 67 years, and the majority of patients were women (*n = *150, 73%). 37 patients (18,3%) were diagnosed with PPPD according to diagnostic criteria. 165 patients (81.7%) did not have PPPD, the most common diagnoses were benign paroxysmal positional vertigo (BPPV) (*n = *44, 21.8%), vestibular migraine (VM) (*n = *33, 16%), Menière’s disease (MD) (*n = *10, 5.0%). 46 patients (22.8%) had other episodic vestibular syndromes, this included patients with orthostatic dizziness (*n = *10, 5%), dizziness related to neck pain/movements without other diagnoses (*n = *8, 4%), dizziness and headaches that did not fulfill criteria for vestibular migraine (*n = *5, 2%) and 1 patient had episodic dizziness due to anxiety. 16 patients (8%) had other chronic vestibular syndromes including 9 patients (4%) describing chronic neck-problems, 5 (2%) with chronic headaches. 8 patients (4%) had other diagnoses including motion-sickness, visual disturbances, central nystagmus, and gait disorder due to gonarthrosis. 8 patients (4%) were diagnosed with acute vestibular syndrome, these were patients with acute onset of dizziness which were improving or resolved. Two of these patients had experienced two episodes of acute vestibular syndrome. Mean duration of dizziness in the PPPD group vs. non-PPPD group was 20 months (SD 22.6, range 3–107) vs. 42 months (SD 68.6, range 3–449); this difference was not statistically significant (*p* = 0.063). Overview of demographics and outcome measures between the PPPD group and the non-PPPD group is shown in Table 1.

**Table 1:** Comparison of demographics, RAND-12, DHI and HADS score in the PPPD group and the non-PPPD group

Characteristic	PPPD	BPPV	Vestibular migraine	Menière’s disease	Non-PPPD (all other diagnoses combined)
*n*	37 (18.3)	44 (21.8)	33 (16.3)	10 (5.0)	165 (81.7)
Women	29 (78.4)	35 (79.6)	28 (84.9)	7 (58.3)	121 (73.3)
Age, years	44.1 (10.1)	50.1 (12.0)	40.4 (13.2)	52.4 (12.1)	46.0 (12.8)
Duration, months	20.0 (22.6)	29.1 (39.7)	59.1 (91.3)	60.2 (78.2)	42.8 (68.6)
DHI	49.2 (17.8)	25.2 (17.8)	38.7 (22.2)	33.4 (15.3)	30.8 (20.3)
RAND-12 physical	39.0 (8.4)	47.1 (11.1)	43.4 (10.5)	50.0 (9.6)	44.6 (10.9)
RAND-12 mental	42.3 (8.8)	48.4 (11.3)	45.9 (10.8)	53.2 (4.7)	46.9 (11.0)
HADS total score	10.3 (5.6)	7.1 (5.9)	9.5 (7.1)	4.9 (3.4)	8.2 (6.5)

Values are absolute frequencies for categorical variables (percentage) and mean for continuous variables (standard deviation)

*PPPD* persistent postural-perceptual dizziness, *DHI* dizziness handicap inventory, *HADS* hospital anxiety and depression scale

### Disease specific QoL (DHI)

**Fig. 1:**
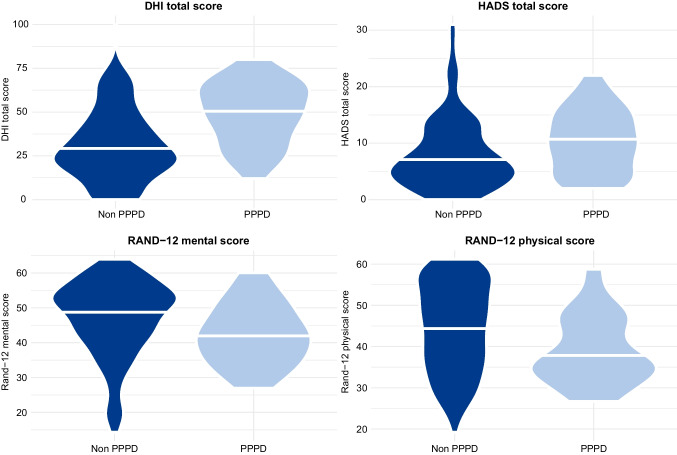
Violin plot illustrating the distribution of DHI score, HADS total score and RAND-12 scores (physical and mental domain separate) between the PPPD group and the non-PPPD group. The mean is illustrated with a white line

Mean DHI total score in the whole study cohort was 34.2 (SD 21.01, range 0–100). Mean DHI total score in the PPPD group was 49.2 (SD 17.8) and was significantly higher compared to the non-PPPD group with mean DHI total score of 30.8 (SD 20.3), *p* < 0.001 (Fig. 1). After adjusting for age and gender we found that patients in the PPPD group had a 17.96 (95% CI (confidence interval) 10.8–25.1); *p* < 0.001) point higher total score on DHI compared to the non-PPPD group. When also adjusting for HADS score, the PPPD group showed a 15.32 (95% CI 8.82–21.8; *p* < 0.001) point higher total score on DHI compared to the non-PPPD group. Regression analyses on DHI total score and RAND-12 scores (physical and mental separate) is shown in Table 2. Distribution of DHI total score, RAND-12 scores and HADS total score between patients with PPPD and patients without PPPD is illustrated in Fig. 1.

**Table 2:** Regression analyses on DHI, physical and mental RAND-12 for patients with PPPD and patients without PPPD

Dependent variable	Characteristic	Unadjusted model		Model adjusted for age and gender		Full model adjusted for age, gender and HADS score	
		Coefficient (95% CI)	*p* value	Coefficient (95% CI)	*p*-value	Coefficient (95% CI)	*p* value
DHI	PPPD	18.34 (11.2, 25.47)	** < 0.001**	17.96 (10.8, 25.1)	** < 0.001**	15.32 (8.82, 21.8)	** < 0.001**
	Age	− 0.16 (− 0.40, 0.08)	0.183	− 0.12 (-0.35, 0.20)	0.278	0.01 (− 0.19, 0.22)	0.913
	Female	3.69 (− 2.99, 10.38)	0.277	2.87 (− 3.45, 9.19)	0.372	3.18 (− 2.51, 8.88)	0.272
	HADS	1.50 (1.09, 1.90)	** < 0.001**			1.38 (0.89, 1.78)	** < 0.001**
RAND-12 physical	PPPD	− 5.63 (− 9.41, − 1.86)	**0.004**	− 5.54 (− 9.32, − 1.75)	**0.004**	− 4.00 (− 7.36, − 0.65)	**0.020**
	Age	0.005 (− 0.12, 0.12)	0.933	− 0.01 (− 0.13, 0.11)	0.899	− 0.09 (− 0.19, 0.02)	0.112
	Female	− 2.34 (− 5.73, 1.05)	0.175	− 2.13 (− 5.47, 1.22)	0.211	− 2.31 (− 5.25, 0.63)	0.123
	HADS	− 0.80 (− 1.01, − 0.59)	** < 0.001**			− 0.80 (− 1.01, − 0.60)	** < 0.001**
RAND-12 mental	PPPD	− 4.59 (− 8.42, − 0.76)	**0.019**	− 4.30 (− 8.11, − 0.48)	**0.027**	− 1.85 (− 4.37, 0.66)	0.148
	Age	0.12 (0.00, 0.24)	0.050	0.11 (− 0.01, 0.23)	0.069	− 0.01 (− 0.09, 0.07)	0.730
	Female	− 1.97 (− 5.39, 1.45)	0.259	− 1.69 (− 5.06, 1.68)	0.325	− 1.98 (− 4.18, 0.23)	0.079
	HADS	− 1.29 (− 1.43, − 1.14)	** < 0.001**			− 1.28 (− 1.43, − 1.12)	** < 0.001**

Significant *p* values are printed in bold

*95% CI* 95% confidence interval, *DHI* dizziness handicap inventory, *PPPD* persistent postural-perceptual dizziness, *HADS* hospital anxiety and depression scale

We also performed a backwards stepwise logistic analysis for the prediction of PPPD with variables from DHI. Here, we found that it is only item 4: “Does walking down the aisle of a supermarket increase your problems?” (OR 1.48; 95% CI 1.16–1.89; *p* = 0.002) and item 7: “Does you problem interfere with your job or household responsibilities?” (OR 1.49; 95% CI 1.12–1.99; *p* = 0.006) that are significant predictors for being diagnosed with PPPD.

When performing regression analyses that were adjusted for age, sex and the various diagnoses we found that PPPD was associated with 19.9 points increase in DHI (95% CI 4.92–34.8; *p* = 0.009) (Table 3).

**Table 3:** Regression analyses on DHI, adjusted for age, gender and diagnoses

	Coefficient (95% CI)	*p* value
Age	− 0.05 (− 0.28, 0.17)	0.640
Gender	1.37 (− 4.92,7.65)	0.668
Diagnoses		
Acute vestibular syndrome	− 17.0 (− 36.1,2.04)	0.080
Meniéres disease	4.63 (− 13.6,22.8)	0.500
Vestibular migraine	9.10 (− 6.05,24.3)	0.237
BPPV	− 3.84 (− 18.7,11.0)	0.610
PPPD	19.9 (4.92,34.8)	**0.009**
Other episodic syndromes	1.74 (− 12.9,16.3)	0.814
Other chronic vestibular syndromes	9.81 (− 6.81,26.4)	0.246

Significant *p* values are printed in bold

*95% CI* 95% confidence interval, *DHI* dizziness handicap inventory, *BPPV* benign paroxysmal positional vertigo, *PPPD* persistent postural-perceptual dizziness

### Health-Related QoL (HRQoL)

Mean RAND-12 physical score in the PPPD group was 39.0 (SD 8.44) vs. 44.64 (SD 10.92) in the non-PPPD group (*p* = 0.004) (Fig. 1). In regression analyses of the RAND-12 physical domain adjusted for age, gender and HADS score, having PPPD was associated with a 4.00 (95% CI − 7.36 to − 0.65; *p* = 0.020) points lower score compared to the patients without PPPD. The effect of a one-point increase in HADS on physical RAND-12 in unadjusted analysis was -0.80 (95% CI − 1.01 to − 0.59; *p* < 0.001). When adjusting for PPPD, age and gender, a one-point increase in HADS was associated with a 0.80 (95% CI − 1.01 to − 0.60; *p* < 0.001) point lower score on the physical RAND-12.

Mean RAND-12 mental score in the PPPD group was 42.33 (SD 8.84) vs. 46.93 (SD 11.03) in the non-PPPD group (*p* = 0.019) (Fig. 1). In regression analyses adjusted for age, gender and HADS there was no significant effect of PPPD on the RAND-12 mental score. The effect of a one-point increase in HADS on RAND-12 mental in unadjusted analysis was − 1.29 (95% CI − 1.43 to − 1.12; *p* < 0.001), and after adjusting for PPPD, age and gender this effect was − 1.28 (95% CI − 1.43 to − 1.12; *p* < 0.001).

Mean HADS total score in the whole study cohort was 8.59 (SD 6.40; range 0–31). Mean HADS total score in the PPPD group was 10.29 (SD 5.61) vs. 8.21 (SD 6.52) in the non-PPPD group (p-value = 0.073).

## Discussion

This study shows that patients with PPPD have a decreased QoL in both a physical and a disease specific domain, compared to patients with other dizziness diagnoses**.** It also shows that PPPD is a different entity than anxiety and/or depression in patients with dizziness.

### Prevalence

In our study population, 18.3% of patients were diagnosed with PPPD, and women were overrepresented in both groups (78.4% in PPPD vs. 73.3% in the non-PPPD group). We did not find a significant difference in age between the two groups.

Considering that PPPD is a relatively new and redefined diagnosis, there is little previous data on the epidemiology of PPPD. According to the consensus document for classification of vestibular disorders of the CCBS from 2017, most epidemiologic data on PPPD is estimated from PPV, SMD, VV, CSD and other functional vestibular diseases. The incidence of PPPD is mostly derived from prospective studies on patients with acute or episodic vestibular disorders (i.e. MD and BPPV) followed for 3–12 months, and these estimates suggests that one out of four patients may develop persistent VV or PPPD-like symptoms after recovery from the initial disorder [3]. In another study by Axer et al. from 2020, 305 (46.6%) of 657 participants met the criteria for PPPD [21]. Our study found a lower prevalence of PPPD in a population of patients with chronic dizziness examined at the ENT-clinic. As the study by Axer et al. found that most of the PPPD patients were young, we do not believe that the exclusion of patients over the age of 67 in our study can explain this difference in prevalence. However, the study design was different, and patients were included in this study consecutively regardless of diagnosis, and there are probably differences in referral practices between different regions and countries. It is however evident that PPPD is highly relevant and a common diagnosis in clinics treating and examining patients with dizziness.

### Disease specific QoL (DHI)

The PPPD group had a significantly higher DHI total score than the non-PPPD group (49.2 vs. 30. 8, *p* < 0.001). This effect was significant even after adjusting for age, gender, and HADS score, which may indicate that there are aspects of PPPD that is not grasped by the HADS scale. We also found that that DHI alone has a significant bearing on whether a patient has PPPD or not, and that the items in the DHI regarding everyday responsibilities and walking down the aisle of a supermarket (i.e. visual trigger) are the most important variables in predicting PPPD.

Two previous studies have found a mean DHI total score among PPPD-patients of 48- 53 which is comparable to our findings [22, 23]. The reason for an increased DHI in the PPPD group is probably complex and multifactorial, but some aspects can presumably be found in the diagnostic criteria themselves [3]; symptoms of dizziness are present on most days, last for long periods of time and are triggered by every-day activities and events that are difficult to avoid (i.e. upright posture, active or passive motion and exposure to moving visual stimuli or complex visual patterns). In addition, PPPD is a chronic disorder with indefinite duration, uncertain pathophysiology and few well-documented treatment regimens [21].

Our findings also coincide well with previous studies showing a correlation between a high DHI total score and psychiatric comorbidity, in particular anxiety and depression [20, 24, 25]. In our study, both groups had an elevated HADS score with mean being 8.59 –suggestive of anxiety and/or depression [19]. The PPPD group had a higher HADS total score than the non-PPPD group, but the difference was not significant (10.29 vs. 8.21, *p* = 0.053). This is an interesting finding, as several items in the DHI are related to anxiety and depression, but the difference in DHI between the groups was still significant after adjusting for HADS, supporting the fact that PPPD is separated from psychiatric disorders, as stated in the consensus document of the CCBS on diagnostic criteria for PPPD [3].

### Health-related QoL (RAND-12)

The PPPD group had a significantly lower RAND-12 physical score than the non-PPPD group (39.0 vs. 44.6; *p* = 0.004), and the significant difference was persistent even after adjusting for age, gender and HADS total score. This indicates that patients with PPPD have a significantly lower physical HRQoL than other dizzy patients. When assessing the physical RAND-12, it is important to mention that our study has not addressed the presence of comorbidity or other variables that may influence physical health negatively, which in turn may affect the physical RAND-12 outcome. We believe that the increased burden of dizziness (DHI) and thus the negative impact on daily life for PPPD patients are important contributors to the reduced HRQoL for these patients.

Patients in the PPPD group had a significantly lower RAND-12 mental score compared to the non-PPPD group, also after adjusting for age and gender, indicating a reduced HRQoL for PPPD patients. The association was not significant after adjusting for age, gender and HADS. HADS and the mental dimension of RAND-12 correlate strongly and are related to similar measures. As previously mentioned, both groups had an elevated HADS total score, suggesting the presence of anxiety and/or depression, which in turn affects the QoL negatively. Several researchers have investigated the causal relationship between psychiatric disorders and dizziness with varying results; Staab et al. found that in 132 patients, 33% of patients had anxiety disorders as the sole cause of dizziness, 34% had neurotologic conditions exacerbating preexisting psychiatric disorders and 33% had neurotologic conditions triggering new anxiety or depressive disorders [26]. On the contrary, Best and colleagues found no difference on incidence rates of psychiatric disorders before and after onset of dizziness [24].

The diagnostic criteria [3] states that PPPD is not a psychiatric condition, which is similar to our findings suggesting that PPPD is a different entity than anxiety and depression. When assessing our findings regarding the RAND-12, it is important to emphasize that there are no benchmarks for minimal clinically important difference for RAND-12 scores [27]. Our study offers an understanding of PPPD as an independent group among the dizzy population, and an insight to the consequences on daily life and QoL for these patients*.*

### Study limitations

Our study population only include patients between 18 and 67 years, and the results are therefore not necessarily applicable to the elderly population. Several studies on chronic dizziness and QoL in patients aged ≥ 60 years have showed an increased burden of dizziness and reduced QoL [6, 28] compared to the younger population, but the impact of PPPD in the elderly population should also be addressed in the future.

Another limitation with the current study is that diagnoses were revised retrospectively to be in accordance with the ICD-11. However, these diagnoses were based on the records from a thorough evaluation by an otolaryngologist, physiotherapist, objective testing and patient reported questionnaires. However, even in a cohort of chronic patients 8 patients were diagnosed with acute vestibular syndrome. These patients had experienced acute onset of vertigo or dizziness, but were improving. However, one could argue that they by definition should have been classified as chronic vestibular syndrome if their symptoms lasted longer than 3 months.

Our study is a cross-sectional study and therefore does not provide information about the causal relationship or causality as to why certain individuals develop PPPD.

### Conclusion

This study demonstrates that PPPD is a common condition among patients with dizziness and that these patients have a significantly higher burden of dizziness (DHI) and a significantly reduced physical HRQoL compared to other dizzy patients. The difference is evident also after adjusting for anxiety and depression, illustrating how PPPD is a different entity from other common psychiatric disorders.

## Data Availability

Local data protection regulations does not allow data to be made available in a public data repository.
